# Ceria-Based Dual-Phase Membranes for High-Temperature Carbon Dioxide Separation: Effect of Iron Doping and Pore Generation with MgO Template

**DOI:** 10.3390/membranes9090108

**Published:** 2019-08-26

**Authors:** Albert Gili, Benjamin Bischoff, Ulla Simon, Franziska Schmidt, Delf Kober, Oliver Görke, Maged F. Bekheet, Aleksander Gurlo

**Affiliations:** 1 Fachgebiet Keramische Werkstoffe / Chair of Advanced Ceramic Materials, Institute of Materials Science and Technology, Faculty III–Process Sciences, Technische Universität Berlin, Hardenbergstr. 40, 10623 Berlin, Germany; 2 Division 5.4 Ceramic Processing and Biomaterials, Bundesanstalt für Materialforschung und -prüfung, Unter den Eichen 44-46, 12203 Berlin, Germany

**Keywords:** samarium doped ceria, SDC, FSDC, CO_2_ separation membranes, scavenging effect of iron, permeability

## Abstract

Dual-phase membranes for high-temperature carbon dioxide separation have emerged as promising technology to mitigate anthropogenic greenhouse gases emissions, especially as a pre- and post-combustion separation technique in coal burning power plants. To implement these membranes industrially, the carbon dioxide permeability must be improved. In this study, Ce_0.8_Sm_0.2_O_2−δ_ (SDC) and Ce_0.8_Sm_0.19_Fe_0.01_O_2−δ_ (FSDC) ceramic powders were used to form the skeleton in dual-phase membranes. The use of MgO as an environmentally friendly pore generator allows control over the membrane porosity and microstructure in order to compare the effect of the membrane’s ceramic phase. The ceramic powders and the resulting membranes were characterized using ICP-OES, HSM, gravimetric analysis, SEM/EDX, and XRD, and the carbon dioxide flux density was quantified using a high-temperature membrane permeation setup. The carbon dioxide permeability slightly increases with the addition of iron in the FSDC membranes compared to the SDC membranes mainly due to the reported scavenging effect of iron with the siliceous impurities, with an additional potential contribution of an increased crystallite size due to viscous flow sintering. The increased permeability of the FSDC system and the proper microstructure control by MgO can be further extended to optimize carbon dioxide permeability in this membrane system.

## 1. Introduction

Climate change is one of the biggest challenges that mankind must face during the 21st century: to achieve sustainable development, anthropogenic CO_2_ emissions must be reduced [1]. Use of dual- phase (DP) membranes for CO_2_ separation in coal-burning power plants was first suggested by Chung et al. as a post-combustion, pre-combustion decarbonization or in the oxyfuel process [2]. A molten carbonate phase was embedded inside the pores of a porous stainless-steel matrix, allowing selective separation of CO_2_ and O_2_. Soon after, the metal support was substituted by an oxygen ion conductive ceramic [3], which allows the selective separation of CO_2_ via its reaction with a lattice oxygen of the ceramic phase. Subsequently, the so formed carbonate ion is transported through the molten carbonate phase via convective flow toward the CO_2_ lean side of the membrane. After inverse reaction and CO_2_ desorption, the oxygen anion is transported back through the ceramic matrix. This ambipolar transport of CO_3_^2−^ and O^2−^ results in a theoretical infinite selectivity toward CO_2_. Modelling work suggests that the overall permeation rate is controlled by the oxygen ion conductivity of the ceramic material [4,5]. In other words, to maximize the CO_2_ flow, efforts should be invested toward increasing the ceramic’s oxygen conductivity and/or decreasing the membrane thickness. More recently, DP membranes have been suggested as membrane reactors for different processes: the water gas shift reaction to produce syngas [6] and in the catalytic ethane-to-ethylene conversion [7].

It is generally accepted that a eutectic mixture of Li, Na, and K carbonates must be used due to its lower melting point (397 °C) and improved stability [8]. Regarding the ceramic phase, several materials have been tested: La_0.6_Sr_0.4_Co_0.8_Fe_0.2_O_3−δ_ (LSCF) [3,9], La_0.85_Ce_0.1_Ga_0.3_Fe_0.65_A_l0.05_O_3−δ_ (LCGFA) [10], Y_0.16_Zr_0.84_O_2−δ_ (YSZ) [8,11,12], Ce_0.9_Gd_0.1_O_2−δ_ (CGO) [8,13,14], Bi_1.5_Y_0.3_Sm_0.2_O_3−δ_ (BYS) [11,15] and Ce_0.8_Sm_0.2_O_2−δ_ (SDC) [16,17]. Generally applied to any high-temperature application and specifically to the DP membranes, stability is a key factor. Several of the above-mentioned materials have shown unstable performance due to different reasons, including phase transformations [15], reaction between CO_2_ and the ceramic phase [9] and irreversible reaction of the carbonate and the ceramic phase [8]. Amongst the several material candidates, SDC has shown high and a remarkably stable CO_2_ permeability [17].

Fluorite-type CeO_2_ can be doped using di- or tri-valent metals, which result in the generation of oxygen vacancies in the ceramic material, which act as a pathway for O^2−^ diffusion [18]. For ionic conductivity in a ceramic material, the overall resistance can be separated into grain and grain boundary resistance. For CeO_2_ and doped-CeO_2_, the contribution of the grain boundary resistance is higher at intermediate temperatures mainly due to the introduction of impurities containing silicon or aluminum [19]. Doping of CeO_2_ with Fe is carried out in order to scavenge the grain boundary impurities and thereby improve the O^2−^ conductivity [18,19,20], although an excess of Fe results in decreased conductivity due to the formation of a Fe_2_O_3_ phase [20]. The exact mechanism of scavenging is not known, although the following options have been suggested [18]: (1) reaction between the scavenger and the silicon-containing impurity, (2) formation of a faceted structure at the grain boundary induced by the scavenger and (3) de-wetting of the silicon containing impurity toward the triple point junction. Fe is also known to decrease the densification temperature of the SDC system [19,20] by inducing viscous flow sintering [21]. Use of sacrificial materials, e.g., NiO template [16], allows for control of the pore volume, size, connectivity, and tortuosity, having a strong influence on the CO_2_ permeability [22]. Other studies relied on the use of MgO as a sustainable, safer and cheaper alternative to metal-containing templates as a hard template for Ag–CGO porous cermets for solid oxide fuel cells [23]. MgO, besides being a less hazardous chemical than NiO, demands no reduction step prior to leaching (which is performed using acetic acid). The simplified procedure to use this pore generator results in a reduction of the final cost of the membrane preparation and the avoidance of using strong acids and hazardous compounds.

The main goal of this work was to study the applicability of iron-doped SDC as a ceramic skeleton for the DP membrane system. Therefore, DP membranes using SDC and FSDC ceramic powders as the oxygen ion conductive phase, a eutectic mixture of lithium, sodium, and potassium carbonates as the carbonate-ion conductive phase and MgO as pore generator were prepared. The powder composition and sintering behavior were characterized using ICP-OES, HSM, and XRD, and the membrane’s morphology and microstructure were characterized using XRD combined with Rietveld refinement, gravimetric analysis and SEM/EDX. The membranes CO_2_ permeability was quantified using a high-temperature membrane permeation setup.

## 2. Materials and Methods 

### 2.1. SDC-FSDC/MgO/MC Membrane Preparation

Doped-CeO_2_ ceramic powders were synthesized using the liquid citrate method reported previously [18,24,25]. Depending on the desired composition, the nitrate precursors (0.25 mol basis): Ce(NO_3_)_3_·6H_2_O (Alfa Aesar, 99.5%, Haverhill, MA, USA), Sm(NO_3_)_3_·6H_2_O (Sigma Aldrich, 99.9%, Saint Louis, MO, USA), and Fe(NO_3_)_3_·9H_2_O (Riedel de Haën, 99.95%, Seelze, Germany) were dissolved in 600 mL of deionized water and mixed with a 100% excess of citric acid (C_6_H_8_O_7_, Roth, 99.5% anhydrous, Karlsruhe, Germany). The mixture was stirred continuously and heated to the boiling point and held under reflux for 4 h. Afterwards, the boiling solution was uncovered, allowing evaporation of the solvent, and thereby promoting gelation. Once the gel was formed, the mixture was dried for 24 h in air at 110 °C. Self-ignition was carried out by placing the gel inside an oven at 410 °C with additional air supply. The resulting powder was ground with a mortar and pestle and further sintered at 550 °C in air for 10 h, with a heating rate of ΔT = 3 °C min^−1^. Prior to its use, the MgO powder (Magnesia 295, Magnesia GmbH, Lüneburg, Germany) was sintered at 1400 °C for 10 h with heating and cooling rates of 2 °C min^−1^ to minimize changes in porosity and pore size of the later composite doped-CeO_2_/MgO membranes. To prepare the doped-CeO_2_/MgO mixtures, powders in a volumetric ratio of doped-CeO_2_:MgO = 70:30 were introduced into a milling vessel with zirconia balls at a ball to powder mass ratio of 20 and rolled on a cylinder for 24 h at 40 rpm. At this point, the powder mixture was granulated by pre-pressing uniaxially into pellets and grinding the resulting disks through a metal sieve. Variable amounts of the granulated powders were placed in stainless-steel molds (diameter ranging from 10–14 mm), which were uniaxially pressed in a Paul-Otto Weber GmbH (Remshalden, Germany) press at room temperature at 6.4 × 10^8^ Pa (for the SDC) and 1.5 × 10^8^ Pa (for the FSDC) for 5 min. A slightly lower pressure was chosen for the FSDC system due to the reported promotion of densification in this system induced by Fe [19]. This procedure was chosen to minimize the difference in the secondary porosity—which results from voids between the ceramic skeleton particles after pressing and not originating from the template removal—between the two materials systems. After pressing, the ceramic disks were sintered for 20 h at 1000 °C using heating rates of 1.5 °C min^−1^. The MgO phase was leached by suspending the pellets using a platinum gasket in an acetic acid (Rotipuran 100%, Roth, Karlsruhe, Germany) aqueous solution (50 wt% in deionized water) at 80 °C under mechanical stirring for 24 h. Afterwards, the membranes were washed with deionized water and allowed to dry in air.

The carbonate phase was introduced in the pores of the pellets after MgO leaching by direct infiltration of molten carbonate, as previously described by Chung et al. [2]. The eutectic mixture of Li_2_CO_3_, Na_2_CO_3_, and K_2_CO_3_ (all carbonates from Merck, Kenilworth, NJ, USA) of 42.5/32.5/25 mol% with a melting temperature of 397 °C [26] was introduced inside a crucible. The membranes to be infiltrated were supported on a platinum gasket attached to a moving alumina cylinder, which was fixed above the carbonate-containing crucible. To prevent any thermal shock on the membrane, a N20/HS oven from Naber (Nordhorn, Germany) was used in conjunction with a system to allow the immersion of the membranes without opening the oven. This facilitated the slow immersion of the membranes inside the molten carbonate and subsequently allowed them to be lifted after the infiltration process. We selected an infiltration temperature of 580 °C (ΔT = 2 °C min^−1^) and a soaking time of 60 min, which resulted in the proper density and viscosity of the molten carbonate to completely infiltrate the membrane’s pores. After cooling, the membranes were removed from the platinum gasket and carefully polished using SiC paper and ethanol to remove excess surface carbonate; the usage of water was avoided to prevent the dissolution of the infiltrated carbonates.

### 2.2. Characterization Methods

Element composition of the SDC and FSDC powders and nitrate precursors was measured by inductively coupled plasma optical emission spectroscopy (ICP-OES) using a Horiba Scientific ICP Ultima2 (Horiba, Kyoto, Japan). Prior to analysis, the ceramic powders were digested in an autoclave using HNO_3_ at 200 °C for 5 h.

The sintering behavior of the SDC and FSDC powders was assessed by hot stage microscopy (HSM), performed with a Hesse instrument (Hesse Instruments, Osterode am Harz, Germany) using uniaxially pressed 3 × 3 mm (w × h) cylinders, operating at a heating rate of 7 °C min^−1^ from room temperature to 1500 °C and an isothermal period of 1 h. The plotted data displays the decrease in area of the laterally observed cylinders with increasing temperature and time. 

The porosity (*ϕ*) of the pellets and membranes was obtained using Equation (1) and Equation (2). The relative error of the porosity calculation is 1%.

(1) $$\phi =1-\left(\frac{m_{m}}{\rho_{c}^{}\cdot V_{m}^{}}\right)$$

(2) $$\rho_{c}=\frac{M_{c}\cdot Z}{N_{A}\cdot a^{3}}$$

Symbols and units for all equations are displayed in the table at the end of the manuscript. Lattice parameters of the ceramic phases were obtained from Rietveld refinement of the XRD patterns, as described subsequently in the results and discussion.

The volumetric fraction of infiltrated carbonate was calculated using Equation (3):

(3) $$X_{MC}=\frac{\rho_{c}-\frac{m_{m}}{V_{m}}}{\rho_{c}-\rho_{mc}}$$

To calculate the ratio of leaching of the MgO phase, comparison of the real (measured) and the theoretical weight of the membrane (assuming complete removal of the template) was done, taking into account that the volumetric ratio to ceramic powder was known (doped-CeO_2_:MgO = 70:30), and its relative error is below 1%. Theoretical weight was calculated using Equation (4):

(4) $$m_{theor}=\frac{m_{bl}\cdot 0.7\cdot \rho_{c}}{0.7\cdot \rho_{c}+0.3\cdot \rho_{MgO}}$$

The ratio of impregnation of the MC phase was calculated using Equation (5), and its relative error is 3%.

(5) $$r_{MC}=\;1-\frac{\phi_{a.l.\;}-\;X_{MC}}{\phi_{a.l.\;}}$$

$\rho_{MgO}$ was obtained from ref. [27].

X-ray diffraction (XRD) was conducted using a Bruker D8 diffractometer operating with a CoK_α1_ radiation source. Measurements were performed between 2*θ* angles of 10–100, with a step size of 0.01719° and 3 seconds per step. The resulting patterns were analyzed and plotted using Origin 2018 software (OriginLab Corporation, Northampton, MA, USA). Crystallite size and lattice parameters were obtained by Rietveld refinement performed using FULLPROF program [28] and profile function 7 [29] (Thompson–Cox–Hastings pseudo-Voigt convoluted with axial divergence asymmetry function). The resolution function of the instrument was obtained from the structure refinement of the LaB_6_ standard. More information regarding the use of the pseudo-Voigt function to calculate the crystallite size and its standard deviation is reported elsewhere [30].

The morphology and element distribution of the pellets and membranes were characterized by SEM/EDX using a LEO Gemini 1530 from Carl Zeiss™ AG (Jena, Germany) at an energy of 5–10 kV. Samples were pre-coated with carbon prior to scanning electron microscopy (SEM) and energy-dispersive X-ray spectroscopy (EDX) to prevent the effect of charging on the samples.

### 2.3. Membrane Performance

Permeation tests were performed between 500 °C and 900 °C using a high-temperature membrane permeation setup, of which the central instrument was a Probostat ® from NorEcs (Oslo, Norway). In-Flow® Mass-flow controllers (MFCs) from Bronkhorst High-Tech BV (AK Ruurlo, The Netherlands) precisely delivered the desired flow rate of He, Ar gases (both from Air Liquide, Alphagaz 99.999 mol%, Paris, France) and CO_2_ (Air Liquide, 99.995%, Paris, France). The almost identical kinetic diameters of Ar (340 pm [31]) compared to CO_2_ (330 pm [32]) encouraged its use as diluent gas to enable better quantification of permeated CO_2_ due to leaks and cracks. Two PXM409 pressure indicators from Omega (Norwalk, CT, USA) indicated pressure in both feed and permeate side. Ceramic disks were fixed on top of an alumina tube using silver rings to seal the membrane. This ensured therefore the separate feeding of the feed and sweep sides. He in the sweep side was used to remove the permeated CO_2_ and ensure a constant partial pressure of CO_2_ across the membrane. The Probostat® was heated using a Heraeus ROPR 5/40 (Hanau, Germany), controlled by an Eurotherm 2404 PID controller. The permeate stream of the setup was analyzed using a GSD-320 O1 mass spectrometer (MS) from Pfeiffer Vacuum. Once the setup was assembled, a heating rate of 1–2 °C min^−1^ was applied under a constant total flow of 10 STP mL min^−1^ in both membrane sides: pure He and a 1:1 mixture of CO_2_:Ar were used. The system was slowly heated up to the softening temperature of the sealing (950 °C), and the Ar concentration was monitored with the MS. Once the Ar concentration in the permeate side dropped, the membrane was properly sealed, and the system was subsequently cooled down to 900 °C. The flow rates in both sides of the membrane were increased to 50 STP mL min^−1^ with the same gas ratios to quantify the flux density of the membranes.

The real flux density of CO_2_ was calculated using the following formulae, based the method previously reported [16]. Total CO_2_ flux (Equation (10)) density is separated between real (Equation (6)) and leakage flux (Equation (7)), which is correlated to the Ar flux (Equation (9)) corrected by the relationship between inlet flows Equation (8), equal to 1 in this study). Permeability of a membrane at a specific temperature is calculated using Equation (11). At each temperature point, the system was allowed to reach steady state before quantifying the flux density, which was multiplied by the membrane thickness to allow comparison between the different doped-CeO_2_/MC membranes. Symbol meaning and units can be found in the abbreviations table at the end of the manuscript. Origin 2018 software was used to plot the results. The relative experimental error of the CO_2_ flux density is 4%.

(6) $$J_{CO_{2},real}=J_{CO_{2},total}-J_{CO_{2}leak}$$

(7) $$J_{CO_{2},leak}=m\cdot J_{Ar,leak}$$

(8) $$m=\frac{Q_{CO_{2},inlet}}{Q_{Ar,inlet}}$$

(9) $$J_{Ar,leak}=\;\frac{X_{Ar,\;permeate}}{1-X_{Ar,permeate}-X_{CO_{2},permeate}}\cdot \frac{Q_{sweep}}{A}$$

(10) $$J_{CO_{2},total}=\;\frac{X_{CO_{2},\;permeate}}{1-X_{Ar,permeate}-X_{CO_{2},permeate}}\cdot \frac{Q_{sweep}}{A}$$

(11) $$K=\;\frac{J_{CO_{2},real}\cdot t}{∆P_{CO_{2}}}$$

## 3. Results

### 3.1. Elemental and Phase Composition and Sintering Behavior of Membrane Materials

Table 1 shows the composition of the SDC and FSDC powders to demonstrate the desired ceramic material composition. For each component (except O) the atomic ratio average and standard deviation are calculated using three different powder batches. As depicted in Table 1, the desired powder compositions were achieved. Moreover, to demonstrate the presence of silicon-containing impurities believed to hinder the grain boundary oxygen conductivity, silicon wt% was also determined. Significant amounts of silicon were observed, and the huge standard deviations clearly showed that silicon contamination was not constant. The metal-precursor nitrate reactants were also analyzed with the same technique to identify the source of Si-contamination and, except for the Ce-nitrate, significant amounts of Si in the range of 0.006–0.013% (wt%) were found. The significant difference between the Si-wt% in the SDC and FSDC powders suggests a strong contribution of other sources of Si-contamination like the furnace refractories [19] or contaminated instrumentation during membrane processing. These findings demonstrate the need to use Fe as a scavenging agent with silicon-containing impurities to improve the grain boundary conductivity of the membrane’s ceramic skeleton.

**Figure 1:**
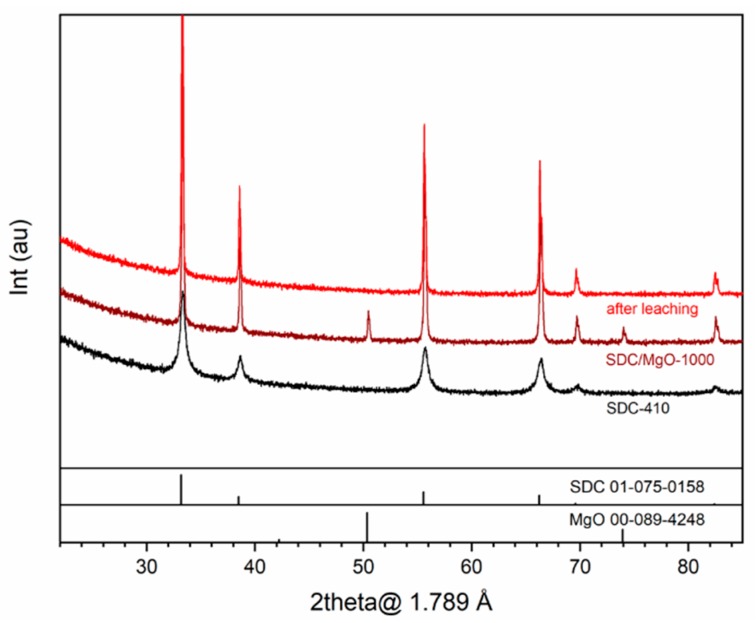
XRD patterns of the (**i**) SDC powders after self-ignition at 410 °C (bottom, black), (**ii**) SDC/MgO pellet after sintering at 1000 °C (middle, maroon) and (**iii**) same pellet after MgO leaching (top, red). Patterns are stacked.

Figure 1 shows the XRD patterns of an SDC material along the preparation process: (1) SDC powder after self-ignition, (2) SDC/MgO pellet after sintering at 1000 °C and (3) the same pellet after leaching of the MgO template. A plot containing XRD patterns of the same membrane preparation steps using the FSDC material as ceramic skeleton is provided in Figure 1, displaying the same trends as the SDC material, and the common observations between the two materials are detailed together. All reflexes belong to a cubic fluorite (Fm-3m) Ce_0.8_Sm_0.2_O_1.9_ (PDF 01-075-0158), with no additional phase. After sintering with the MgO pore generator at 1000 °C, both material pellets logically display additional MgO reflections (PDF 00-089-4248). Moreover, a decrease in the width of the XRD reflections corresponding to the SDC phase is observed after sintering, which suggests an increase in the crystallite size with the sintering temperature. After leaching (top patterns in both figures), the MgO reflexes disappear from the pellet surface without altering the SDC fluorite structure. For the FSDC system, the lack of any FeO_x_ crystal phase detection at the Fe concentration under study matches the previously reported lower limit of appearance of Fe_2_O_3_ at Fe 1.5% atomic [20], which would have a detrimental effect on the ceramic phase conductivity.

**Figure 2:**
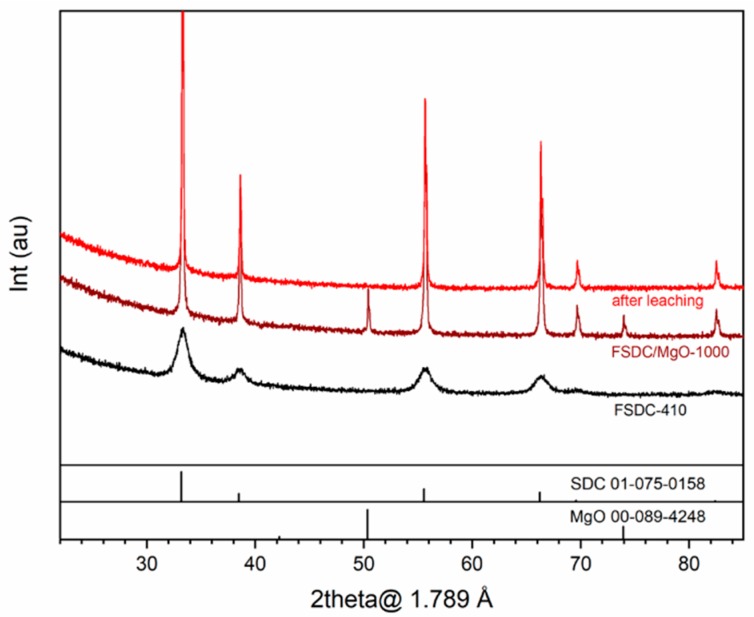
XRD patterns of the (**i**) FSDC powders after self-ignition at 410 °C (bottom, black), (**ii**) FSDC/MgO pellet after sintering at 1000 °C (middle, maroon) and (**iii**) same pellet after MgO leaching (top, red). Patterns are stacked.

To evaluate the effect of Fe addition on the lattice parameter and the effect of the sintering temperature and Fe addition on the crystallite size of the FSDC material, Rietveld refinement of the XRD data displayed in Figure 1 and Figure 2 was performed and is shown in Table 2. The addition of Fe to the SDC system results in a slight decrease in the lattice parameter of the FSDC after self- ignition from 5.4376 (s = 0.0005) to 5.4331 (s = 0.0009) Å, indicating the partial substitution of the larger Sm^3+^ atoms (coordination VIII, 1.079 Å [33]) by the smaller Fe^3+^ (coordination VIII, 0.78 [33]). This contraction in the unit cell combined with the absence of additional iron oxide phases unambiguously points to the formation of a solid solution (composition shown in Table 1). After sintering at 1000 °C, the FSDC lattice parameter slightly increases to 5.4343 (s = 0.0001), suggesting the ex-solution of Fe from the ceramic material [18]. Finally, after leaching, the lattice parameter of both materials match at 5.4353 (s = 0.0001), in good agreement with the reported lattice parameter of SDC [34,35,36]. Application of this lattice parameter to Equation (2) results in bulk densities of 7.138 g cm^−3^ and 7.081 g cm^−3^ for the SDC and FSDC powders, respectively. Fe addition results in an smaller crystallite size of the FSDC compared to that of the SDC after self-ignition, as previously observed [18], which can be attributed to the decrease in the interfacial contact area of the crystallites during the self-ignition step [18]. After sintering at 1000 °C, crystallite size increases for both materials due to sintering, the crystallite size of the FSDC (88.9 nm) material being slightly higher than that of SDC (80.9 nm) due to the viscous flow sintering. Finally, after leaching, the crystallite size of both materials increases to above 100 nm.

**Figure 3:**
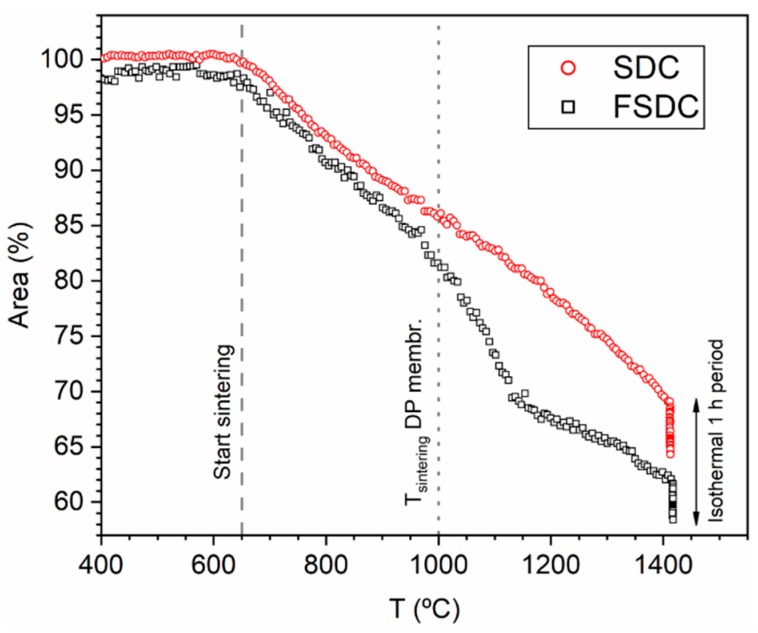
Sintering behavior of the SDC and FSDC powders characterized by HSM. Dashed line highlights the start of the sintering for both materials. Dotted line shows the sintering temperature of the later DP membranes.

Figure 3 shows the comparison between SDC and FSDC change in area as function of temperature obtained by HSM. Both powder systems begin to sinter at 650 °C and follow similar trends up to temperatures of 950 °C, in which the FSDC powder displays an increased change of area. This rapid sintering behavior is due to the viscous flow sintering induced by the Fe addition as previously reported [18]. At 1050 °C, the change in area of the FSDC further increases compared to the SDC system, the later showing a rather constant change rate all along the temperature range. These results suggest not to sinter the pellets at temperatures above 1000 °C to minimize the effect of the viscous flow in the FSDC, which could result in critical microstructure differences and consequently not allow comparison of the two materials as DP membranes. Lower sintering temperatures compromise the pellets mechanical stability and were therefore disregarded.

### 3.2. Porosity and Element Distribution of the Sintered Membranes

The porosities of SDC and FSDC pellets during the different membrane preparation steps are shown in Table 3. Both pellets display similar porosities despite the enhanced sintering of the FSDC samples due to viscous flow sintering [18], demonstrating the proper pore-generator effect of the MgO sacrificial template and the beneficial effect of applying different pressures during pellet pressing. Therefore, the effect of the different porosities (and subsequently of the ratio between ceramic phase and carbonate phase) is minimized, enabling direct comparison of the two materials as ceramic skeletons for DP membranes. As seen, the MgO template is almost completely removed by leaching in both ceramic systems without compromising the membrane’s mechanical stability, demonstrating the applicability of the suggested synthesis route and template material to the DP system. The percentage of impregnated carbonate was calculated using Equation (5) and is included in Table 3. The impregnation method successfully implements 0.98–1 of the theoretical maximal carbonates for both membrane systems.

**Figure 4:**
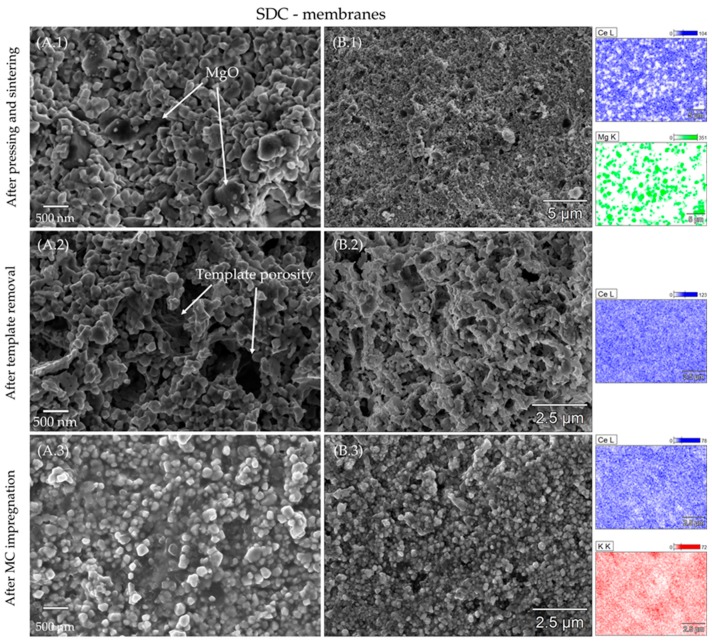
SEM images of an SDC membrane all along its preparation steps: (**1**) after sintering, (**2**) after template removal and (**3**) after MC impregnation. (**A**) column displays SEM imaging, while (**B**) column displays EDX characterization with the correspondent mappings of Ce (blue), Mg (green) and K (red).

**Figure 5:**
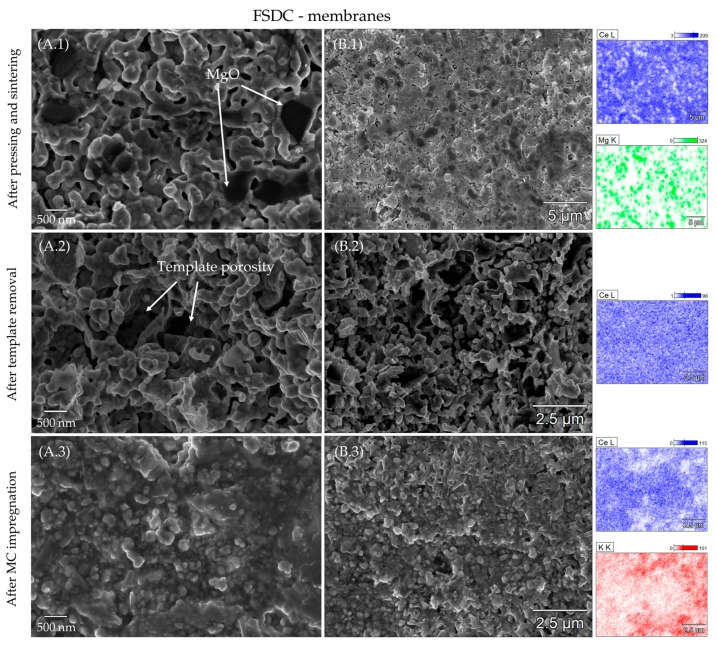
SEM images of a FSDC membrane all along its preparation steps: (**1**) after sintering, **(2**) after template removal and (**3**) after MC impregnation. (**A**) column displays SEM imaging, while (**B**) column displays EDX characterization with the correspondent mappings of Ce (blue), Mg (green) and K (red).

Figure 4 and Figure 5 show the morphology and element distribution as revealed by SEM/EDX characterization of SDC and FSDC membranes all along their preparation steps: (1) after pressing with MgO and sintering, (2) after leaching of the MgO phase and finally (3) after impregnation. For the (1) images, the MgO template is clearly observable before leaching, as highlighted by the difference in particle size and contrast (MgO appears darker compared to the doped-CeO_2_ particles) and by the EDX mapping shown in the right part of Figure 4 and Figure 5 Ce (blue) and Mg (green) mapping clearly demonstrate the separation between the two compounds in the pellet. After leaching, the MgO is removed to a high extent as proved by the lack of MgO particles and the consequent generation of porosity in the pellets. Moreover, no Mg could be detected by the EDX analysis (data not shown), demonstrating the successfully applied leaching step. In the leached pellets, two types of porosity can be observed: a primary porosity derived from the leaching of the template and a secondary porosity derived from the voids between the doped-CeO_2_ particles, the former being predominant. Comparison of A.2 in Figure 4 and Figure 5 demonstrates the soft effect of the increased viscous flow of the FSDC material over SDC induced by Fe at 1000 °C on the membrane microstructure and specially on the secondary porosity, as shown by the similar morphologies of the membranes after leaching. The particle size of both materials was obtained after leaching of the MgO template (A.2 image) using ImageJ [37] software by averaging 50 particles and assuming spherical crystallites. The particle size in the FSDC leached pellets of 156.0 nm (s = 37.5) is higher than the 121.0 nm (s = 30.1) in the SDC pellets. These values suggest that each particle for both systems is composed of one or two crystallites (see Table 2). The results demonstrate the increase in particle size in the FSDC material compared to that of the SDC material caused by the viscous sintering flow. Both the use of MgO and the difference in applied pressure while pellet processing, results in extremely similar membrane microstructure, allowing for a rational comparison between these two materials. After impregnation with the carbonate mixture, the pores of both membrane systems appear completely filled, matching the observation displayed in Table 3. EDX mapping of the B.3 images in both Figure 4 and Figure 5 displays a homogeneous distribution of K (red) and Ce (blue) over the analyzed membrane surfaces. These membranes surfaces had to be polished shortly before SEM characterization due to the re-crystallization of the carbonate species on the surface, most probably induced by moisture in the air and posterior evaporation in the microscope chamber, which appeared to fully cover the membrane’s surface. As is later proved by the permeability tests, this fact did not result in an irreversible coverage of the membrane surface, as the membranes were capable of selectively separating CO_2_ from the feed gas mixture.

### 3.3. Membrane Performance

**Figure 6:**
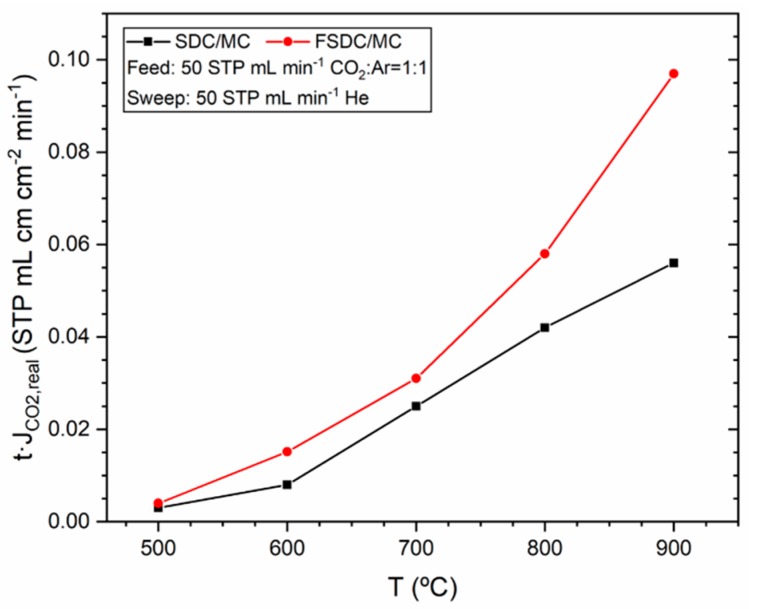
CO_2_ flux density multiplied by membrane thickness as function of the temperature for the SDC and FSDC DP membranes.

The real flux density of CO_2_ multiplied by the membrane thickness (*t·*$J_{CO_{2},real}$) of the resulting SDC and FSDC DP membranes as a function of the temperature is displayed in Figure 6. For both systems, the operating conditions are identical, as described earlier. Lines between the data points have been added for visual clarity. For both materials, the flux density increases with temperature due to the increased oxygen ion conductivity of the ceramic skeleton. The FSDC displays an increased CO_2_ flux density compared to the SDC at all the temperatures tested. The reason is twofold: (1) the Fe scavenging effect on the silicon-containing impurities previously reported [18,19,20], which results in a decrease of the grain boundary resistivity in the doped-CeO_2_ ceramic skeleton, and (2) an increase in the ceramic skeleton crystallite size as displayed in Table 2. An increase in the crystallite size results in a decreased resistance of the membrane to oxygen ion conductivity due to the decrease of the amount of grain boundaries, the main contributor to overall resistivity in doped-CeO_2_ materials [18]. The generation of additional oxygen vacancies by addition of Fe was disregarded as potential contribution to the improved CO_2_ flux density in the FSDC membrane, as Sm^3+^ was substituted by Fe^3+^, and the reported almost identical oxygen vacancy concentration between SDC and 0.5–1.5 FSDC (0.5–1.5 at%) [18].

The CO_2_ permeability of the membranes at 900 °C is 7.59 × 10^−11^ mol m m^−2^ s^−1^ Pa^−1^ (SDC) and 1.31 × 10^−10^ mol m m^−2^ s^−1^ Pa^−1^ (FSDC), below that (1.52 × 10^−10^ mol m m^−2^ s^−1^ Pa^−1^ for SDC) reported previously [17]. The difference can be explained due to: (1) differences in the membrane porosity (36% in ref. [17], taking into account that the CO_2_ flux density decreases with increasing porosity [5]), (2) an increased sintering temperature of the SDC pellets (1100 °C) compared to our study (1000 °C) and (3) different testing conditions. The perm-selectivities of the membranes (CO_2_/Ar) are 141.4 (SDC) and 153.4 (FSDC) at 900 °C and they decrease with temperature due to the relatively lower concentration of CO_2_ in the permeate side, caused by the decrease of the flux density via the selective mechanism with temperature. These values are similar to those previously reported [16,17] and highlight the high selectivity of the DP membrane system. The significant Ar concentration detected in the permeate side (usually in the range of 20–90 ppm) are due to membrane cracks or most probably—as SEM characterization highlighted no visible cracks—imperfect sealings.

**Figure 7:**
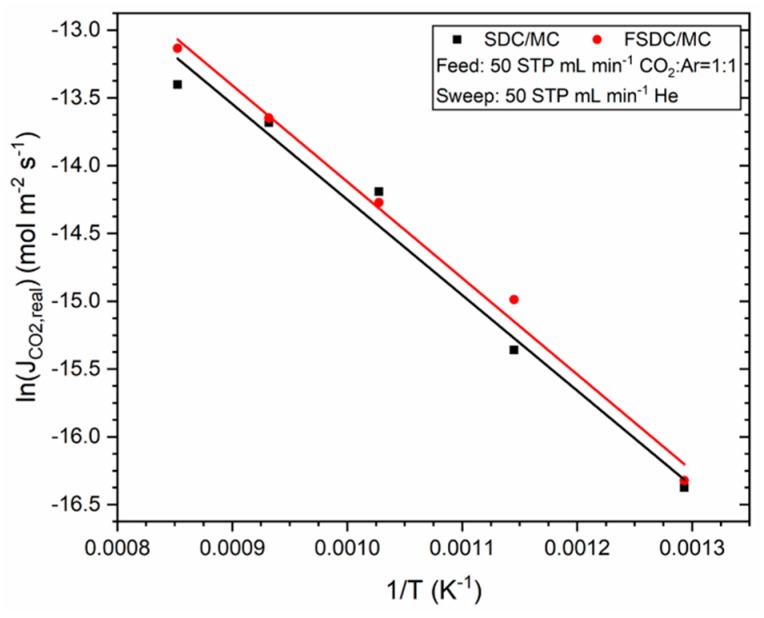
Arrhenius Equation fit displaying the ln of the CO_2_ flux density as function of the inverse of the temperature for the SDC and FSDC DP membranes.

Figure 7 shows the Arrhenius Equation fit for the SDC and FSDC DP membranes experiments shown in Figure 6. The good fittings (R^2^ > 0.98) yield activation energies of 58.6 (for the SDC) and 59.1 kJ mol^−1^ (for the FSDC) membranes. These activation energy values are similar to those previously reported for SDC DP membranes [16,17] and suggest that the ceramic skeleton oxygen conductivity is the rate determining step for the overall permeation. Although we did not quantify the stability of the membrane, previous works suggest that the SDC DP membranes do not deactivate with time [17].

**Figure 8:**
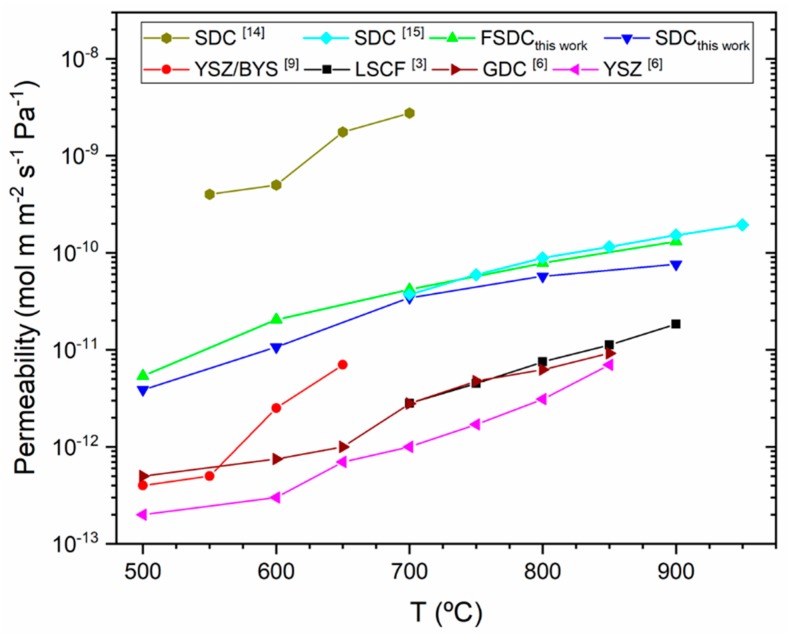
Comparison chart displaying experimental permeability data of several DP membrane systems from literature and this study.

Figure 8 shows a comparison chart including selected permeability values of different DP membrane systems and the values obtained in this work. The permeability data from literature [3,8,11,16,17] was obtained from plotted experimental data (except for [17]) and is therefore not precise. Also, the testing conditions of the different membrane systems differ, including differences in flow rates, CO_2_ partial pressure, and, for the SDC with NiO as pore generator [16], the use of H_2_ in the feed side. Figure 8 serves as a benchmark of our new FSDC DP membrane, suggesting that microstructure optimization can lead to a substantial improvement. The highest permeability displayed by SDC [16] was achieved by use of NiO as pore generator via a co-precipitation method with the SDC ceramic skeleton, which was later sintered at 1400 °C, resulting in highly interconnected 3D channels [16]. Tuning the MgO pore generator particle size, and also investigating the co- precipitation of the MgO and the FSDC materials are expected to allow more precise control of the membrane’s microstructure and improve the CO_2_ permeability.

## 4. Conclusions

SDC and FSDC containing 1.1 at% Fe were successfully synthesized using the liquid citrate method. MgO was successfully applied in order to control the porosity of the SDC and FSDC ceramic pellets. The MgO pore generator is directly (without a reduction step) and easily removed after sintering using acetic acid, as proven by gravimetric study and SEM/EDX characterization, without compromising the membrane’s mechanical stability. After the removal of the pore generator, the similar porosity of the two material systems allows comparison of the two oxygen-ion conductive materials as DP membranes. Successful impregnation of the ceramic skeleton with a eutectic molten carbonate mixture was demonstrated by gravimetric analysis and SEM/EDX characterization.

The addition of iron to the SDC material results in an increase of the viscous flow of the ceramic material demonstrated by HSM and in an increase of the crystallite size after sintering, as demonstrated with XRD combined with refinement of the resulting patterns. SEM characterization demonstrates an increase of the particle size in the FSDC system compared to that of the SDC. The combination of the increase in crystallite size with the documented scavenging effect of iron with silicon-containing impurities results in an increase of the CO_2_ permeability under the conditions tested compared to the iron-free SDC system. A maximum CO_2_ permeability of 1.31 × 10^−10^ mol m m^−2^ s^−1^ Pa^−1^ at 900 °C with equimolar CO_2_:Ar feed was achieved using the FSDC/MC DP membrane. The use of MgO as sacrificial phase can be further extended to study the effect of the porosity and the pore size of the ceramic phase on the CO_2_ permeability in order to optimize this membrane system. Co-precipitation of the template and the doped-CeO_2_ precursors can be studied to further control the microstructure and improve the phase interconnectivity and homogeneity of the mixed powder.

## Figures and Tables

**Table 1:** SDC and FSDC powder composition by ICP-OES.

Sample	Ce_atomic_ (-)	Sm_atomic_ (-)	Fe_atomic_ (-)	Si_wt%_ (wt%)	Formula
SDC	0.810 (s = 0.001)	0.190 (s = 0.005)	0.000 (s = 0.000)	0.088 (s = 0.139)	Ce_0.81_Sm_0.19_O_2-δ_
FSDC	0.797 (s = 0.003)	0.192 (s = 0.003)	0.011 (s = 0.001)	0.255 (s = 0.399)	Ce_0.797_Sm_0.192_Fe_0.011_O_2-δ_

**Table 2:** Lattice parameters and crystallite sizes of the SDC and FSDC powders and pellets obtained from the Rietveld refinement of the XRD patterns.

Sample ^1^	Crystallite Size (nm)	Lattice Parameter (Å)
SDC-410	12.1 (s = 0.2)	5.4376 (s = 0.0005)
SDC-1000-b.l.	80.5 (s = 1.5)	5.4360 (s = 0.0002)
SDC-1000-a.l.	>100	5.4353 (s = 0.0001)
FSDC-410	5.6 (s = 0.1)	5.4331 (s = 0.0009)
FSDC-1000-b.l.	88.9 (s = 1.6)	5.4343 (s = 0.0001)
FSDC-1000-a.l.	>100	5.4353 (s = 0.0001)

^1^ b.l. = before leaching, a.l. = after leaching the MgO phase.

**Table 3:** SDC and FSDC pellets porosity all along the membrane preparation steps and fraction of leached MgO and impregnated carbonate.

Material	Porosity after Sintering (%)	Porosity after Leaching (%)	Leached MgO ^1^ (-)	Impregnated Carbonate ^2^ (-)
SDC-1000	20.3	44.6	0.99	1
FSDC-1000	23.6	47.0	0.99	0.98

^1^ Calculated using Equation (4); ^2^ Calculated using Equation (5).

## List of Abbreviations and Symbols

**Symbol**	**Description**	**Units**
**Abbreviations**		
DP	Dual-phase	-
FSDC	Iron and samarium co-doped ceria	-
HSM	Hot stage microscopy	-
ICP-OES	Inductively coupled plasma optical emission spectrometry	-
MC	Molten carbonate	-
SDC	Samarium doped ceria	-
SEM/EDX	Scanning electron microscopy/energy-dispersive X-ray spectroscopy	-
XRD	X-ray diffraction	-
**Symbols**		
A	Membrane effective area	cm^−2^
a	Lattice parameter	cm or Å
J	Flux density	mL min^−1^ cm^−2^
K	Membrane permeability	mol m m^−2^ s^−1^ Pa^−1^
N_A_	Avogadro’s number	-
P	Pressure	Pa
Q	Volumetric flow rate	mL min^−1^
t	Thickness	mm
X	Component fraction or ratio	-
Z	Formula units per unit cell	-
**Greek letters**		
ρ	Density	g cm^−3^
ϕ	Porosity	-
**Subindex**		
*a.l.*	After leaching	-
*b.l.*	Before leaching	-
*c*	Ceramic phase (SDC or FSDC)	-
*i*	species, usually gas Ar, CO_2_ or He	-
*inlet*	Referring to the feed gas inlet in the membrane system	-
*leak*	Flux density through cracks and defective sealing	-
*m*	Membrane	-
*MC*	Molten carbonate	-
*MgO*	MgO template	-
*permeate*	Referring to the permeate out of the membrane system	-
*real*	Real gas flux density	-
*sweep*	Referring to the sweep gas inlet in the membrane system	-
*theor*	Theoretical mass of the leached membranes	-
*total*	Total flux density (real + leak)	-
